# Distinct Activation of Defense-Related Gene Networks in Rosetted Versus Symptomless Shoots in Rose Rosette Virus-Infected Roses

**DOI:** 10.3390/v18080906

**Published:** 2026-08-18

**Authors:** Shakil Hosain, Venura Herath, Reghan Mutethia, Michael V. Kolomiets, Kevin Ong, Oscar Riera-Lizarazu, Jeanmarie Verchot

**Affiliations:** 1 Department of Plant Pathology and Microbiology, Texas A&M University, 496 Olsen Blvd, College Station, TX 77845, USA; shakil.hosain@tamu.edu (S.H.); reghan.mutethia@tamu.edu (R.M.); mike.kolomiets@ag.tamu.edu (M.V.K.); kevin.ong@ag.tamu.edu (K.O.); 2 Department of Agricultural Biology, Faculty of Agriculture, University of Peradeniya, Peradeniya 20000, Sri Lanka; venura@agri.pdn.ac.lk; 3 Department of Horticultural Sciences, Texas A&M University, 495 Horticulture Rd Suite 202, College Station, TX 77843, USA; oscar.riera-lizarazu@ag.tamu.edu

**Keywords:** *Emaravirus rosae*, rose rosette virus, systemic acquired resistance, antiviral defenses

## Abstract

Rose rosette virus (RRV) causes witches’ broom disease in roses. This study examined whether differences in disease in these spatially distinct domains are associated with local changes in defense-related hormones and transcriptional reprogramming. The levels of defense-related phytohormones salicylic acid (SA), jasmonic acid (JA), 9-lipoxygenase (9-LOX), 13-lipoxygenase (13-LOX), and derived oxylipins were different in these distinct tissues. We curated differentially expressed genes involved in the biosynthesis, storage, and signaling of SA, JA, and oxylipins to reveal how specific defense pathways correlate with these distinct disease states. Gene ontology (GO) analysis and the Disease Resistance Analysis and Gene Orthology 3 (DRAGO3) pipeline revealed stress-associated genes and classical resistance (R) gene families that were differently expressed in these distinct tissue domains. Promoter analysis of defense-associated transcription factors and histone modifiers revealed hormone responsive elements, suggesting complex hormone crosstalk is involved in defense-related gene expression. We confirmed by RT-qPCR, a notable subset of defense genes in the chromosome 5 hotspot, specifically in the linkage group 5 (LG5) quantitative trait loci, linked to reduced RRV susceptibility, was altered in both field-infected and greenhouse-inoculated plants. The association of high versus low disease states with distinct defense hormone signatures and gene expression patterns suggests that local immune states, rather than whole-plant defenses, determine whether a cane becomes rosetted or remains asymptomatic.

## 1. Introduction

*Emaravirus rosae* (rose rosette virus; RRV) is a negative-sense RNA virus that causes rosette disease, characterized by abnormal clustering of branches, hyperthorniness, as well as malformed leaves and flowers near the growing tips of the shoot [1,2]. Two eriophyid mite species (*Phyllocoptes fructiphilus* Keifer and *P. arcana*) are common vectors for RRV and this virus/vector pair originally spread throughout North America with a very susceptible host, *Rosa multiflora* [3,4,5,6]. RRV and its vector continue to spread into landscape roses, causing devastation to landscapes and botanical gardens across North America [1].

RRV is a significant economic threat to landscape and garden roses, which hold substantial cultural and commercial value worldwide [7,8,9,10]. The interaction between RRV, its mite vector, and roses presents a unique model for studying plant–virus interactions, particularly given the complex architecture of roses and the distinct disease phenotype. Rose plants have several thick main stems emanating from the base of the plant, called strong canes. Younger lateral branches grow yearly from the canes that may or may not continue to expand. A notable feature of RRD is the occurrence of contrasting symptomatic and asymptomatic conditions in different canes and branches within the same host plant. New evidence indicates that asymptomatic tissues show significantly lower virus levels than symptomatic tissues, making investigations of RRV infection in roses a model system to dissect differential gene expression and hormonal dynamics that can promote or suppress infection [11,12,13]. Such studies are invaluable for uncovering genes and pathways that contribute to either susceptibility or resistance to viral pathogens.

The identification of roses with field resistance to RRD requires laborious field-based screening trials where scientists introduce large numbers of viruliferous mites to increase the disease pressure, a process known as augmentation. Breeders and geneticists typically assess RRD resistance by quantifying the number of plant shoots or branches with reduced rosetting symptoms and testing for the presence of RRV in comparison to susceptible varieties [14]. Using genetic mapping populations, multiple quantitative trait loci (QTL) associated with partial resistance to RRD were identified on several chromosomes (also called linkage groups, LGs) numbered 1, 3, 5, 6, and 7, with the largest effect attributed to LG5. A meta-analysis combining diploid and tetraploid datasets was used to refine the genetic intervals on LG5 for three meta-QTLs: MetaRRD5.1, MetaRRD5.2, and MetaRRD5.3 [13,15,16]. A candidate gene search identified putative disease resistance genes and factors known to contribute to antiviral defenses [16]. Efforts are now underway to leverage known sources of resistance to breed new cultivars with enhanced RRD resistance and combine them with best management practices to disrupt disease spread and safeguard the long-term production of one of the most important plants for the floriculture industry [12,14].

Mutethia et al., (2025) provided foundational transcriptomic analysis of genes associated with architectural changes in RRV-infected rose plants (var. Double Knockout) by comparing asymptomatic and symptomatic tissues collected from the same plants [11]. This prior study showed that hormone-driven signaling pathways, including auxin, gibberellins, abscisic acid, and strigolactones signaling, were differentially altered in symptomatic relative to asymptomatic tissues. Building on these observations, the present study conducts a focused secondary analysis of the published RNA-seq dataset to generate hypotheses about whether defense-associated signaling networks differ between tissues with low virus accumulation (asymptomatic) and high virus accumulation (symptomatic). We hypothesize that the lower viral levels in asymptomatic tissues indicate more effective antiviral resistance in these shoots and branches relative to symptomatic tissues. Specifically, we evaluated differential expression of genes associated with pathogen recognition, cell wall and plasma membrane signaling, phytohormone and oxylipin pathways, chromatin modification, and defense-related transcriptional regulation in leaf tissues collected from symptomatic and asymptomatic canes of the same field-grown rose shrubs. The shrubs were naturally infected with RRV by its eriophyid mite vector and did not receive augmentation treatment. Rather than establishing a causal mechanism of antiviral resistance or symptom development, this analysis provides a hypothesis generating framework that prioritizes defense-associated pathways and candidate genes for functional validation in rose. These research outcomes hold substantial importance toward enhancing our understanding of plant defense mechanisms in roses.

## 2. Materials and Methods

### 2.1. Sample Preparation and Hormone-Related Metabolite Measurement

Sample extraction was performed using 100 mg of tissues from six infected and six healthy rose plants. Symptomatic and asymptomatic tissues were collected from different canes of the same plants [11] and homogenized using 500 µL of phytohormone extraction buffer (1-propanol/water/HCl, 2:1:0.002 *v*/*v*/*v*) containing 500 nM isotopically labeled internal standards [dddJA (2,4,4-d3; acetyl-2,2-d2 JA; CDN Isotopes, Pointe-Claire, QC, Canada), and dddSA (d6-SA; Sigma-Aldrich, St. Louis, MO, USA)] and then agitated for 30 min at 4 °C before adding 500 µL dichloromethane. Samples were again agitated for 30 min at 4 °C, and then centrifuged at 21,000× *g* for 10 min. The lower organic layer was transferred to 2 mL glass vials (Restek Corp., Bellefonte, PA, USA) and evaporated by N_2_ gas. Dried samples were resuspended in 150 µL LC-grade methanol and transferred to 1.7 mL tubes and placed at −20 °C for 48 h. Following filtration with 0.22 µm filters (Sigma-Aldrich, St. Louis, MO, USA), samples were placed in glass inserts (Restek Corp., Bellefonte, PA, USA) and stored in 2 mL glass vials at −20 °C until run on the LC-MS/MS. A 10 µL aliquot of each sample was injected into a Triple Quad™ 4500 LC-MS/MS system (SCIEX, Framingham, MA, USA) using electrospray ionization (ESI) in negative ion mode with multiple reaction monitoring. Chromatography was performed using an Ascentis C18 column (3 cm × 2.1 mm, 2.7 µm; Sigma-Aldrich, St. Louis, MO, USA). The mobile phase gradient was set at a 0.495 mL/min flow rate consisting of solution A (0.02% [*v*/*v*] acetic acid in water) and solution B (0.02% [*v*/*v*] acetic acid in acetonitrile) with the following gradient (time in minutes -% [*v*/*v*] solution B): 0.5–10%, 1.0–20%, 21.0–70%, 24.6–100%, 24.8–10%, 29-stop. The metabolites listed in Table S1 were quantified using Analyst v.1.7.2 software by comparison of internal standards.

### 2.2. Statistical Analysis of Hormone-Related Metabolites and Data Visualization

The FASTER cluster, hosted by the Texas A&M University High-Performance Research Computing Center, was used for performing statistical analysis in R (ver. 4.4.2). We evaluated salicylic acid (SA), jasmonic acid (JA), and oxylipin metabolite concentrations to determine both the global variance and pairwise differential abundance across healthy, asymptomatic, and symptomatic tissues (Figure S1). Our sequential analytical strategy was designed to handle the specific biological and statistical subtleties of our metabolite dataset, namely, the presence of both highly abundant compounds and trace signaling molecules across a relatively small sample size (*n* = 6 per condition).

The non-parametric Kruskal–Wallis test was used to screen the raw metabolite concentrations across healthy, asymptomatic, and symptomatic tissues (*n* = 6). This rank-based test was used to identify metabolites whose distributions differ among the three conditions without assuming normally distributed data. Metabolites with significant overall Kruskal–Wallis results (*p* < 0.05) were aggregated in Table S1.

We transformed the raw concentration data of the selected metabolites into log_2_ scale to stabilize variance across the dataset and to quantify pairwise differential abundance (Symptomatic vs. Healthy, Asymptomatic vs. Healthy, and Symptomatic vs. Asymptomatic). We applied the limma empirical Bayes framework (ver. 3.62.1) in RStudio (ver. 4.4.2) to log2-transformed data specifically to resolve pairwise contrasts (healthy vs asymptomatic, healthy vs. symptomatic, and asymptomatic vs. symptomatic) [17] which uses an empirical Bayes moderated *t*-test to fit linear models to the transformed data. We obtained Log_2_ Fold Changes (Log_2_FC) and corresponding *p* values for each pairwise contrast to evaluate magnitude and directionality of the hormonal (metabolite) shifts.

To visualize systemic hormone-related metabolite shifts across conditions, a hierarchical clustering heatmap was generated using the pheatmap package (v1.0.12) in R. The heatmap was restricted to metabolites that showed statistically significant differential abundance identified by the limma analysis (FDR-adjusted *p* < 0.05). To enable direct side-by-side visual comparison of relative changes across metabolites with substantially different baseline concentration scales, Log_2_(x + 1)-transformed values were standardized using row-wise Z-scores.

### 2.3. Plant Material, cDNA Library Construction, Illumina Sequencing

Samples were collected from ten rosetting and asymptomatic canes of field-grown rose shrubs (var. Double Knockout) in Texas USA (lat. 31.56 N, long. −97.14 W). Ten samples of healthy rose shrubs (var. Double Knockout) were collected from another Texas (USA) field plot (lat. 30.62 N, long, 96.33 W). Total RNA extraction was performed using the Maxwell^®^ RSC Plant RNA Kit (Promega Corp. Madison, WI, USA). RNA samples were pooled and then divided into 5 aliquots of 200 ng. Endpoint RT-PCR was performed using RRV-F/R primer pair to confirm infection [18]. This approach to RNA sequencing using pooled samples to identify differentially expressed genes has been shown to yield reliable results in plant studies [18,19]. High-throughput mRNA sequencing was performed using the Illumina NovaSeq platform (Novogene Corp., Sacramento, CA, USA) using a paired-end 150 bp sequencing strategy for the cDNA libraries. Read trimming and quality control were performed with fastp and FastQC, respectively [20,21]. RNA-seq data is accessible through the NCBI Sequence Reach Archive (BioProect PRJNA1285908).

### 2.4. RNA-Seq Read Mapping and Transcript-Level Expression Analysis

HISAT2 2.2.1 was used to align reads to the reference genome, *Rosa chinensis* ‘Old Blush’ homozygous genome v2.0 [22,23]. SAMtools was then used to convert files into BAM format [24]. StringTie was used on each sample’s BAM file to assemble transcripts, estimate their abundance [25], and produce a Ballgown-style directory [26]. Finally, prepDE.py was used to convert the StringTie abundance/coverage estimates into both gene- and transcript-level count matrices (csv files), which were imported into DESeq2 for count-based differential expression analysis [11]. The healthy RNA-seq dataset was used as a background standard to compare RRV effects between asymptomatic and symptomatic tissues, and lists of differentially expressed genes (DEGs) used in this study are provided in Tables S2–S6.

### 2.5. Gene Annotation, Gene Ontology (GO), KEGG Enrichment, and Resistance Genes Analysis

The *Rosa chinensis* (RchiOBHm-v2) genome assembly (www.rosaceae.org) was used to obtain Ensembl and NCBI Gene IDs, GO terms, and InterPro descriptions of protein families, domains, and enzymatic motifs (https://amigo.geneontology.org/amigo/dd_browse; last accessed 28 July 2024). Protein fasta sequences were retrieved for 1903 of the 2000 DEGs (other sequences were putative non-coding RNAs) using the BioMart tool in Ensembl Plants (https://plants.ensembl.org/biomart/martview: Access Date: 27 August 2025) by selecting the *Rosa chinensis* (RchiOBHm-v2) genome assembly.

Functional annotation was performed in OmicsBox v3.4 platform using: (a) the Blast2GO pipeline (BioBam Bioinformatics, Valencia, Spain) [27] with an E-value threshold set to 1.0 × 10^−3^ for protein similarity; (b) BLASTp and InterProScan 5 to identify protein functional domains [28] and (c) GO enrichment analysis, restricting GO terms to level 3 and applying a threshold of *p* < 0.05) (Tables S2–S6; Figure S2). GO enrichment outputs were cross-referenced with previous annotations obtained using ShinyGO (http://bioinformatics.sdstate.edu/go/; last accessed 16 September 2024) and DAVID (https://david.ncifcrf.gov/; last accessed 2 October 2024) [11,29]. KEGG pathway information (map00380, map00908, map00906, map00940, map00592, and map04075) was used for manual biological interpretation.

To identify pathogen recognition genes, protein sequences of the DEGs were submitted to the PRGdb4.0 DRAGO3 pipeline (https://prgdb.org/prgdb4/drago3 last accessed 30 July 2025) [30,31] for analysis against the RchiOBHm-v2 genome assembly. Predictions from DRAGO 3 were further evaluated with PRGminer v1.0.0 using default parameters. Resistance gene predictions with high confidence (>70%) were retained for classification of resistance gene classes [31].

### 2.6. Ab Initio Promoter Analysis

Promoter analysis was performed on a subset of differently expressed genes which we manually identified as key signaling intermediates, transcriptional regulators, and epigenetic/chromatin modifiers within core plant immune signaling networks: NDR1, MAPKKKs, WRKYs, and NACs, PHD histone modifiers, OBERON2, and HDT2 to determine which upstream hormonal signals orchestrate their expression. Promoter sequences of 1000 bp upstream from the transcription start site were extracted from the *R. chinensis* (RchiOBHm-v2) assembly using the BioMart tool from the EnsemblPlants database (last accessed 30 July 2025). *Cis* elements associated with hormone biosynthesis, metabolism, and response were retrieved from the New Place database (https://rapdb.dna.naro.go.jp/PLACE/?action=newplace; last accessed 30 July 2025) [32]. These elements were mapped to the gene promoters using Geneious Prime ver. 2025.0 (https://www.geneious.com).

### 2.7. Rose Plant Inoculations (Var. Double Knockout) Using the RRV-Infectious Clone

Rose liners (pre-rooted cuttings) were obtained from Star^®^ Roses and Plants (West Grove, PA, USA) and grown in a soil mixture consisting of Miracle-Gro^®^ Plant Mix for roses (ScottsMiracleGro, Marysville, OH, USA) and Kellogg^®^ All-Natural Mix for trees, shrubs, and roses (Kellog Garden Products, Carson, CA, USA). At the time of transplanting, 0.25 teaspoon of Osmocote^®^ Smart-Release Plus Outdoor/Indoor All-Purpose Fertilizer (15-9-12 N–P_2_O_5_–K_2_O) (The Scotts Company, Marysville, OH, USA) was incorporated into the growth medium. In addition, plants were fertigated every 14 days with Miracle-Gro^®^ Water-Soluble All-Purpose Plant Food (18–24–16 N–P_2_O_5_–K_2_O) at a rate of 0.5 teaspoon per US gallon of water. Plants were maintained in a greenhouse at 25 °C, with supplemental lighting provided as needed to ensure a 12 h photo period.

The RRV-infectious clone constructs were maintained in *Agrobacterium tumefaciens* strain GV3101 [33,34]. Selected colonies were grown overnight in YEB supplemented with kanamycin and rifampicin (50 µg mL^−1^ each). The cultures were collected by centrifugation at 4000× *g* for 15 min and resuspended in infiltration buffer (0.1 M MgCl_2_, 500 mM MES hydrates, and 100 mM acetosyringone). The agrobacterium cultures were adjusted to OD_600_ 0.3 using the NanoDrop™ 2000 spectrophotometer (ThermoFisher Scientific, Waltham, MA, USA) and incubated at room temperature for three hours in the dark. Equal culture volumes of RRV segments 1–7 were mixed and delivered to fifteen plants using Master Airbrush Model E91 (TCP Global, Duncan, SC, USA). Mock plants were treated with untransformed agrobacterium cultures. Infection was confirmed by endpoint RT-PCR using RRV-F/R primers [18].

### 2.8. Integration of QTL Mapping with Differential Gene Expression Analysis

To identify defense genes within the QTL interval, the physical positions of three meta-QTLs (MetaRRD5.1, MetaRRD5.2, and MetaRRD5.3) on linkage group 5 (LG5) were used to extract the Gene IDs for all genes within the same interval within the *Rosa chinensis* ‘Old Blush’ reference genome v2.0 (GDR; https://www.rosaceae.org) using the MegaSearch tool. Gene lists were downloaded in tabular format (last accessed on 8 September 2024). The QTL gene lists were compared with the DEGs to obtain the final gene list (Table S8).

### 2.9. RT-PCR and RT-qPCR for Assessing Host Gene Expression

Total RNA was extracted from 100 mg leaf samples using the Maxwell^®^ RSC Plant RNA Kit (Promega Corp., Madison, WI, USA). cDNA was prepared using 150 ng RNA pooled from the field-infected plants or 150 ng RNA from individual RRV-inoculated rose plants, random hexamer primers, and Maxima™ H Minus Reverse Transcriptase (ThermoFisher Scientific, Waltham, MA, USA). Endpoint RT-PCR was performed using GoTaq^®^ G2 Flex DNA polymerase (Promega Corp., Madison, WI, USA) [35], and qPCR was performed using PowerUp™ SYBR™ Green Master Mix (ThermoFisher Scientific, Waltham, MA, USA). The qPCR reactions were carried out at an initial holding step at 50 °C for 2 min and 95 °C for 2 min, followed by 40 amplification cycles of 95 °C for 30 s, 57 °C for 30 s, and 72 °C for 1 min. The final extension step was carried out at 72 °C for 2 min. Table S9 lists primers for endpoint and quantitative PCR.

To determine whether statistically significant differences in gene expression existed across the three physiological states, a One-Way Analysis of Variance (ANOVA) was conducted for each gene. For variables demonstrating a significant overall ANOVA result, Tukey’s Honestly Significant Difference (HSD) post hoc test was subsequently performed to evaluate all pairwise group comparisons (healthy vs. asymptomatic, asymptomatic vs. symptomatic, and healthy vs. symptomatic) while adjusting for multiple comparisons. Statistical significance was established at an alpha level of *p* < 0.05. All statistical testing and data wrangling were performed using the R statistical programming environment (v4.5.3) using RStudio (v2024.12.) utilizing the *dplyr* (v 1.2.0) and *tidyr* (v1.3.2) packages. Data visualizations were generated using the *ggplot2* (v4.0.2) package, presenting the mean relative expression levels alongside standard deviation (SD) error bars. To denote statistical significance on the generated bar plots, a compact letter display (CLD) was calculated using the *multcompView* (0.1–11) package.

## 3. Results

### 3.1. The Dynamic Levels of Salicylic Acid (SA), Jasmonic Acid (JA), Oxylipin, and Traumatin Metabolites Associated with RRD

**Figure 1:**
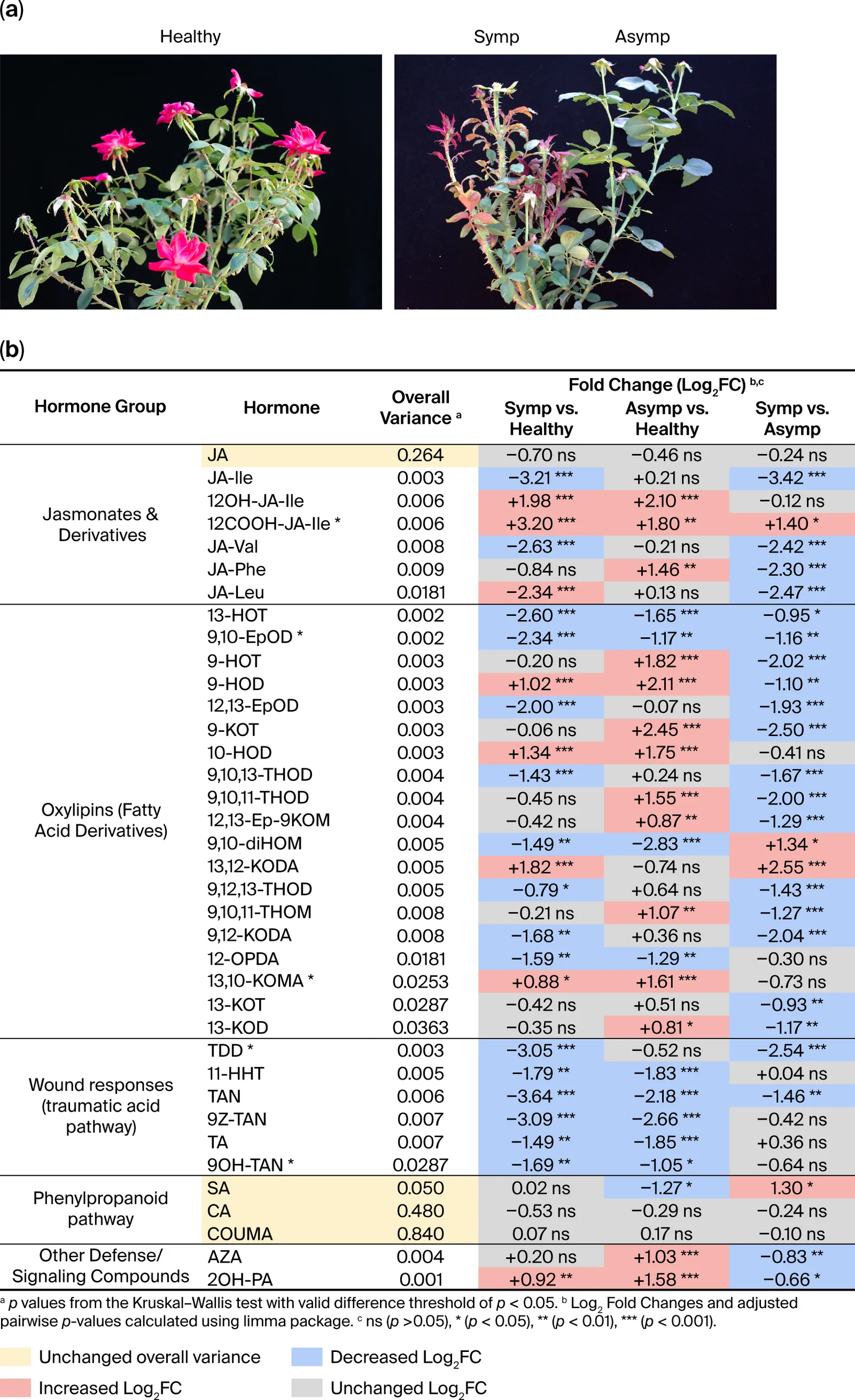
Metabolite analysis performed using healthy and RRV-infected garden rose plants. (**a**) Example of a healthy and RRV-infected garden rose plant used in this study. Separate canes and branches represent symptomatic rosetted tissue and asymptomatic infected tissues. (**b**) Summary table detailing the overall variance (Kruskal–Wallis test) and pairwise Log_2_Fold Changes (limma analysis) for jasmonates, oxylipins, wound responsive traumatins, phenylpropanoids, and other defense/signaling compounds across healthy, asymptomatic, and symptomatic conditions. JA, SA, CA, and COUMA are included in the figure even though they were not significantly altered. See Table S1 for full names and corresponding abbreviations.

Field-grown rose plants became naturally infected by RRV through feeding by their eriophyid mite vector, resulting in uneven virus accumulation in rosetted and asymptomatic tissues (Figure 1a) [11]. To characterize the overall response of the field-grown garden plants, samples representing symptomatic (rosetted), asymptomatic, and healthy tissues were used to obtain an average status of each tissue type and to capture the general “field reality” of how natural infection impacts plant metabolites. Although this approach obscures plant-to-plant variation, it is appropriate for our goal of quantifying plot-level differences among tissue types. We were particularly keen to measure salicylic acid (SA) which is a key antiviral defense hormone [36,37,38] whose synthesis proceeds through several precursors in the phenylpropanoid pathway, including trans-cinnamic acid (CA) and ortho-coumaric acid (COUMA) [39,40,41]. SA is often antagonistic to jasmonic acid (JA), which shapes local defenses, including defenses to insect herbivory, and systemic acquired resistance (SAR). JA biosynthesis proceeds through the 13-lipoxygenase (13-LOX) conversion of α-linolenic acid through several steps, including the intermediate precursor cis-12-oxyphytodienoic acid (12-OPDA) [42]. We also measured intermediates in the lipid-derived 13-LOX and 9-lipoxygenases (9-LOX) pathways (Table S1) which convert fatty acids to bioactive oxylipins that can contribute to local defenses and SAR [43,44,45,46]. Traumatins, which are often induced by wounding, were also examined.

LC-MS/MS profiling quantified 42 metabolites across healthy plants (*n* = 6) and infected plants, from which both symptomatic (*n* = 6) and asymptomatic (*n* = 6) tissues were sampled. These compounds and their abbreviations are listed in Table S1. To assess global, systemic variance across the three conditions, raw concentration data from the individual replicates were subjected to a non-parametric Kruskal–Wallis test. This identified 37 metabolites exhibiting statistically significant systemic variance (*p* < 0.05) (Figure 1b). In contrast, five metabolites, including free JA, SA, CA, and COUMA, showed *p* > 0.05, indicating no significant global shifts in abundance across the conditions. To rigorously evaluate the magnitude and directionality of these shifts despite the small sample size, the data were analyzed using empirical Bayes moderated linear models (limma). This facilitated precise pairwise contrasts of symptomatic versus healthy, asymptomatic versus healthy, and symptomatic versus asymptomatic. The limma analysis revealed clear transitive patterns in hormonal abundance. For instance, when a metabolite was significantly depleted in symptomatic tissues compared to healthy ones, but unchanged in asymptomatic tissues, the direct symptomatic versus asymptomatic comparison predictably mirrored the symptomatic versus healthy profile. A similar, though smaller, magnitude effect was observed for significantly elevated metabolites (Figure 1b).

Interestingly, the bioactive jasmonates, JA-Ile, JA-Val, and JA-Leu, which are important systemic immune signals, exhibited highly significant negative log2FC differences. Rather than accumulating, these critical defense hormones are profoundly depleted in RRV-infected rosetted tissues, suggesting a targeted viral suppression or host-mediated shutdown of bioactive jasmonate signaling (Figure 1b). In contrast, the inactive jasmonate conjugates, JA-Phe, 12OH-JA-Ile and 12COOH-JA-Ile exhibited significant log2FC differences that were either increased or unchanged in symptomatic and asymptomatic tissues relative to healthy tissues, consistent with a shift in jasmonate metabolism toward lower levels of the bioactive forms (Figure 1b).

For 19 oxylipins, the significant log2FC differences between symptomatic and either asymptomatic or healthy tissues pointed to fine-tuning levels of the metabolic pool size, which likely reflects dynamic levels of compounds moving through their respective pathways (Figure 1b). Traumatins were represented in this dataset by six compounds that exhibited significantly lower log2FC differences between symptomatic and healthy tissues, five compounds exhibited elevated differences in asymptomatic compared to healthy tissues, and only two were significantly elevated when comparing symptomatic and asymptomatic tissues (Figure 1b).

SA, a systemic antiviral defense hormone, exhibited significantly lower log2FC differences when comparing asymptomatic and healthy tissues but significantly higher differences when comparing asymptomatic and symptomatic tissues (Figure 1b). Azelaic acid (AzA) is a mobile signal upstream of SA, and the log2FC differences in comparing asymptomatic and healthy tissues were higher or lower in a manner that was opposite of SA levels. Finally, 2OH-PA (2-hydroxy-palmitic acid) belongs to the family of α-hydroxy long-chain fatty acids that serve as acyl chains in plant sphingolipids. Sphingolipids containing 2-hydroxy fatty acids are known to organize plasma membrane nanodomains and to modulate ROS-based immune responses and stress tolerance. Thus, elevated 2OH-PA in symptomatic leaves may reflect changes in RRV-induced membrane lipid remodeling.

### 3.2. Differential Gene Expression Patterns Influencing Defense Hormone Production and Signaling

**Figure 2:**
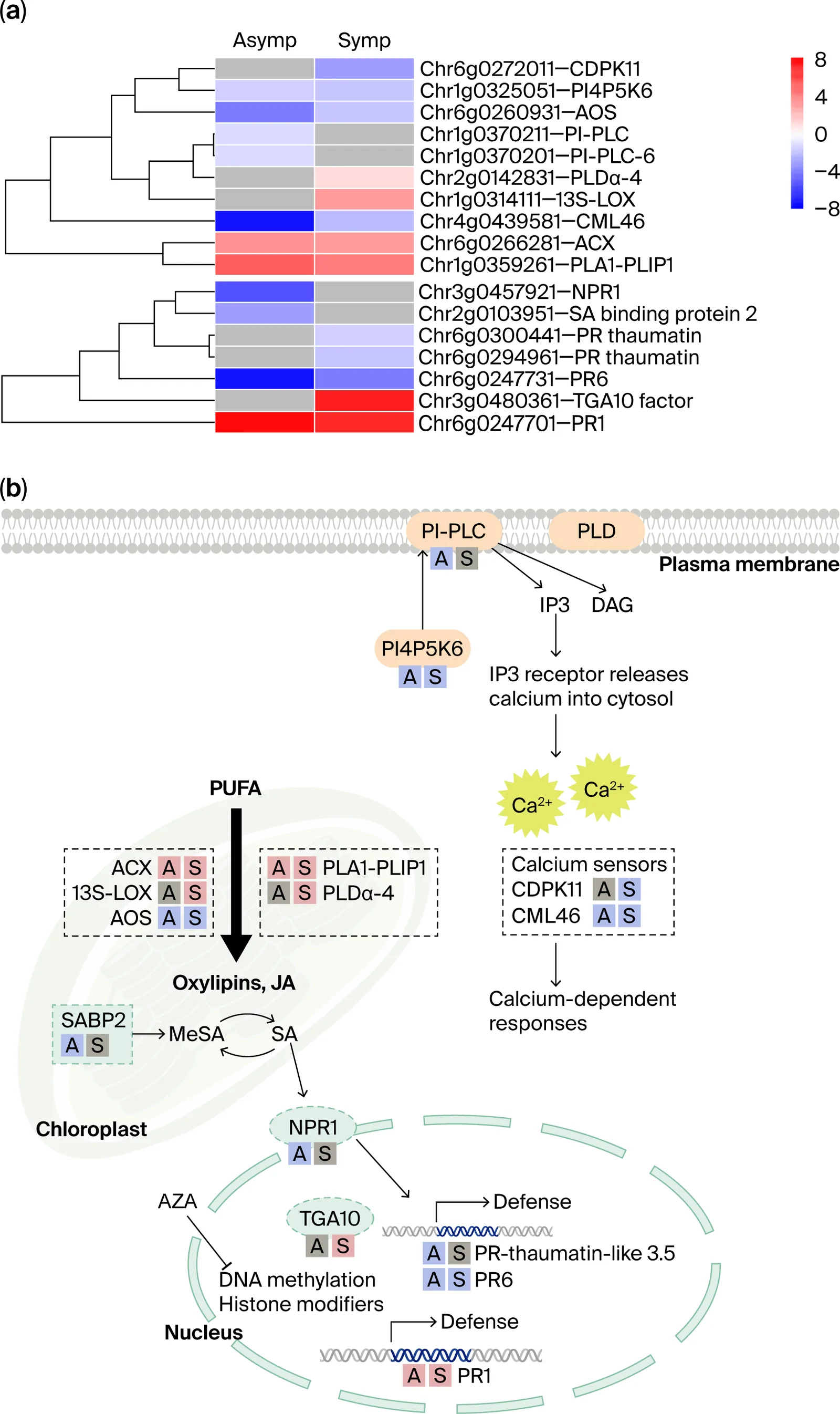
Contrasting transcript levels in symptomatic and asymptomatic tissues of seventeen genes involved in conversion to JA and SA combined with key responsive genes. (**a**) Heatmap showing log_2_ fold changes (log2FC) in the expression of 17 genes, relative to healthy tissues, for asymptomatic (Asymp) and symptomatic (Symp) samples. Only genes with statistically significant changes (*p*adj < 0.05) are shown. Each column represents a condition (Asymp or Symp), and each row represents one gene, with gene names listed on the right. The color scale (right) indicates the magnitude and direction of log2FC values, where red boxes denote increased transcript levels and blue boxes denote decreased transcript levels compared with healthy tissues. Full gene descriptions and detailed statistics are provided in Tables S2–S6. (**b**) Illustration highlights elements of three pathways, also represented by different style arrows. Near the plasma membrane is the plant phosphoinositide-specific phospholipase C (PI-PLC) and phospholipase D (PLD) which are linked to Ca^2+^ regulated defenses. PI-PLC hydrolyzes phosphoinositides to produce inositol phosphates and diacylglycerol (DAG) which is converted to inositol phosphates, which can trigger intracellular calcium release during stress, including pathogen-triggered defense [49,50,51]. PLDα promotes phosphatidic acid production that feeds into the jasmonic acid (JA) biosynthetic pathway [47,48]. In the chloroplast, PLA1-PLIP phospholipases liberate polyunsaturated fatty acids (PUFAs) to supply substrates for oxylipin and JA biosynthesis. Also in the chloroplast, SA is converted to MeSA and activates defense signaling to the nucleus involving SABP2, NPR1, TGA10. AzA blocks histone modification in the nucleus affecting SA-mediated gene expression.

We reanalyzed a pre-existing RNA-seq dataset of RRV-infected plants to determine gene expression patterns associated with SA, JA, and oxylipin pathways in asymptomatic and symptomatic tissues. Differential gene expression analysis revealed that RRV infection perturbed multiple steps in the 13S-LOX biosynthetic pathway, influencing the production and partitioning of bioactive JA (Figure 2a,b). Transcripts encoding chloroplastic phospholipase A (PLA-PLP1; Chr1g0359261), phospholipase D (PLDα-4; Chr2g0142831), acyl-CoA oxidase (ACX; Chr6g0266281), and 13S-LOX (Chr1g0314111) showed a 1- to 5-positive log2FC in asymptomatic and symptomatic tissues compared to healthy leaves (Figure 2a,b, and Tables S3 and S4). This pattern is consistent with an upstream flux into the jasmonate/13-ketols versus other oxylipin branch pathways. We are not certain whether the PLD here is clearly PLDα or another isoform which localize to distinct compartments and are associated with different stress responses [47,48]. PLA1-PLIP phospholipases liberate polyunsaturated fatty acids (PUFAs) such as α-linoleic acid from membranes to supply substrates for oxylipin (including JA) biosynthesis [49].

Interestingly, an *allene oxide synthase* (*AOS*; Chr6g0260931), which encodes a critical bottleneck enzyme for generating the allene oxide precursors of jasmonates [52], was strongly downregulated (log2FC −4.0 and −1.7) in asymptomatic and symptomatic tissues, respectively (Figure 2a,b, and Tables S5 and S6). This severe transcriptional repression of *AOS* provides a direct mechanistic explanation for the profound depletion of downstream bioactive jasmonates (JA-Ile, JA-Val, and JA-Leu) observed in our metabolomic analysis of symptomatic tissues (Figure 1b). Furthermore, this transcriptional bottleneck likely diverts upstream 13-LOX-derived intermediates away from JA synthesis and into alternative oxylipin branch pathways [52]. Genes that are commonly upregulated or downregulated between conditions are presented in Table S7.

To relate these changes to SA signaling and SAR, genes involved in SA-methyl salicylate (MeSA) conversion and downstream SA-responsive transcriptional networks were evaluated (Figure 2a,b, and Tables S3–S7). SA binding proteins (SABP2) function as methyl esterases converting MeSA to SA to fine-tune active SA levels [53,54]. SA activates NPR1, which forms a complex with TGA10-like transcription factors to drive PR1 and other pathogenesis-related (PR) gene expression [55,56,57,58]. *SABP2* (Chr2g0103951) and an *NPR1-like* factor (Chr3g0457921) were strongly downregulated (log_2_FC ≈ −3 and −5, respectively) in asymptomatic tissues but not obviously altered in symptomatic tissues. At the same time, two *PR5-like* (Chr6g0300441 and Chr60294961) were not obviously altered in asymptomatic tissues, although lower in symptomatic tissues (log_2_FC ≈ −1.5 and −1.8, respectively), whereas *PR6* was lower in both symptomatic and asymptomatic tissues (Chr6g0247731; log_2_FC ≈ −7 and −4, respectively). Among the upregulated genes in symptomatic tissues was the SA-related *TGA10* transcription factor (Chr3g0480361; log_2_FC = 6.84), and *PR1* was upregulated in both symptomatic and asymptomatic tissues (Chr6g0247701; log_2_FC = 6.43 and 7.43, respectively). Canonical SABP2 and NPR1 are positive components of SA-dependent SAR, and downregulation of these components would typically be expected to compromise SA-induced expression of *TGA10*, *PR1*, *PR5*, and *PR6*; however, the strong induction of *TGA10* and *PR1* under high virus titer conditions suggests that their activation may occur via SA-NPR1 independent or compensatory pathways.

Because the metabolite data also implicated changes in lipid-derived signaling, genes linked to phosphatidylinositol-specific phospholipase C (PI-PLC) signaling were also evaluated for their differential expression (Figure 2a,b and Tables S3–S7). Phosphatidylinositol 4-phosphate 5-kinase 6 (PI4P5K6) functions at the plasma membrane to enhance the substrate pool for PI-PLC, leading to the elevation in phosphatidic acid (PA) and diacylglycerol (DAG) signals, which act as secondary messengers for calcium-related signaling [59,60,61]. *PI4P5K6* (Chr1g0325051; log_2_FC ≈ −1.7 and −1.6) and *Calmodulin-like 46* (*CML46*; Chr4g0439581; log_2_FC ≈ −2 and −6) were downregulated in both symptomatic and asymptomatic tissues, respectively. Two *PI-PLC* genes (Chr1g0370211 and Chr1g0370201; both log_2_FC ≈ −1) were mildly downregulated in asymptomatic and not significantly altered in symptomatic tissues. In addition to CML46, another calcium sensor linked to PI-PLC activity, *Calcium dependent protein kinase 11* (*CDPK11*; Chr6g0272011; log_2_FC = −3), was also downregulated in symptomatic tissue but not obviously affected in asymptomatic tissues (Figure 2a,b, and Tables S3–S7). Notably, azelaic acid (AzA) fits into this model because it globally reduces cytosine methylation and histone acetylation to de-repress immune or stress-responsive genes. These coordinated changes suggest that, in addition to reprogramming oxylipin, JA, and SA pathways, RRV infection compromised a lipid–calcium signaling layer of systemic resistance in both high and low virus infection conditions.

### 3.3. Gene Ontology Enrichment Analysis Reveals Significant Numbers of Stimuli-Responsive Genes

**Figure 3:**
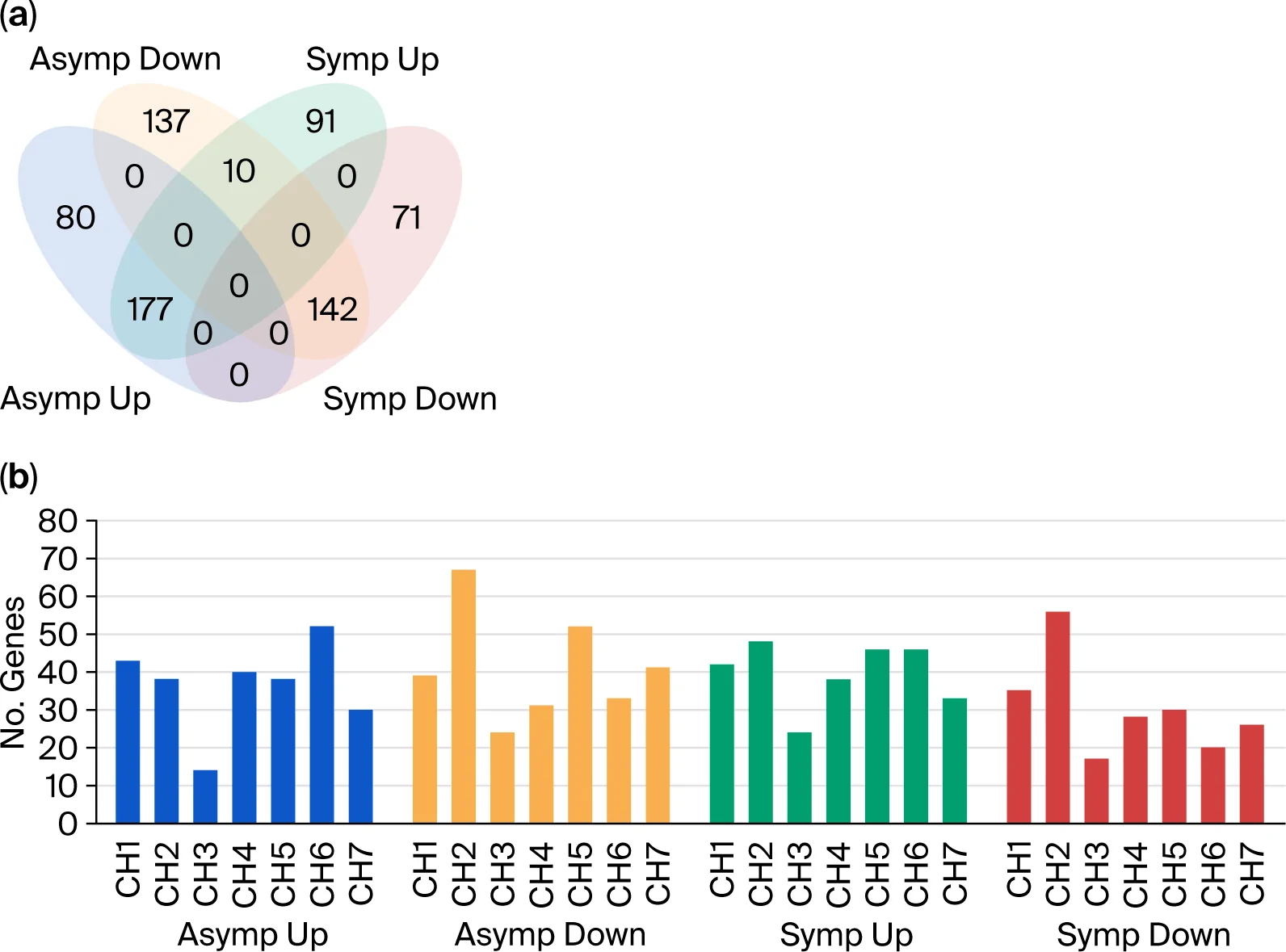
Gene ontology (GO) enrichment analysis of differentially expressed genes related to plant defense and stress responses. (**a**) Venn diagram shows GO-enriched defense- and stress-related genes identified in asymptomatic (Asymp) and symptomatic (Symp) tissues, highlighting the number of genes that are unique to each condition as well as shared between both. Colors used in Venn diagrams distinguish categories of genes. (**b**) Bar chart showing how these GO-enriched genes are distributed across chromosomes, indicating the chromosome locations of defense- and stress-associated loci identified in the transcriptome. Bars are color coded according to the Venn diagram.

Gene ontology (GO) enrichment analysis of the transcriptome dataset [11] revealed eleven GO category terms associated with responses to various external and internal stimuli (e.g., chemical, stress, stimulus, abiotic stress, endogenous stimuli, external stimuli, response to other organism, biotic stress, regulate response to stimuli), as well as signaling and signal transduction processes (Figure S3). A Venn diagram illustrates unique and overlapping gene sets (Figure 3a). In asymptomatic tissues, 257 genes were upregulated, of which 80 were unique to this condition, while 177 were shared with symptomatic tissues. A total of 289 genes were downregulated in asymptomatic tissues, with 137 unique to asymptomatic tissues and 142 shared with symptomatic tissues. Symptomatic tissues exhibited 91 uniquely upregulated and 71 uniquely downregulated genes, and an additional 10 genes showed opposite regulation between the two conditions (Figure 3a). These patterns suggest that while core signaling networks are conserved across conditions, asymptomatic tissues exhibit a distinct subset of stimulus-responsive genes that may contribute to defense priming and lower virus accumulation.

Chromosome mapping was performed to evaluate whether groups of differentially expressed genes, especially those that might be associated with disease resistance or susceptibility, might cluster in certain regions. Interestingly, downregulated genes were overrepresented on chromosome 2 and chromosome 5 (Figure 3b), which is notable because quantitative trait loci for RRD resistance have been mapped to these chromosomes [13,15]. This strengthens the connection between transcriptomic changes and genetic loci that breeders target for increased resistance.

### 3.4. Protein Phosphorylation Networks Are Distinctly Altered in Symptomatic and Asymptomatic Tissues

**Figure 4:**
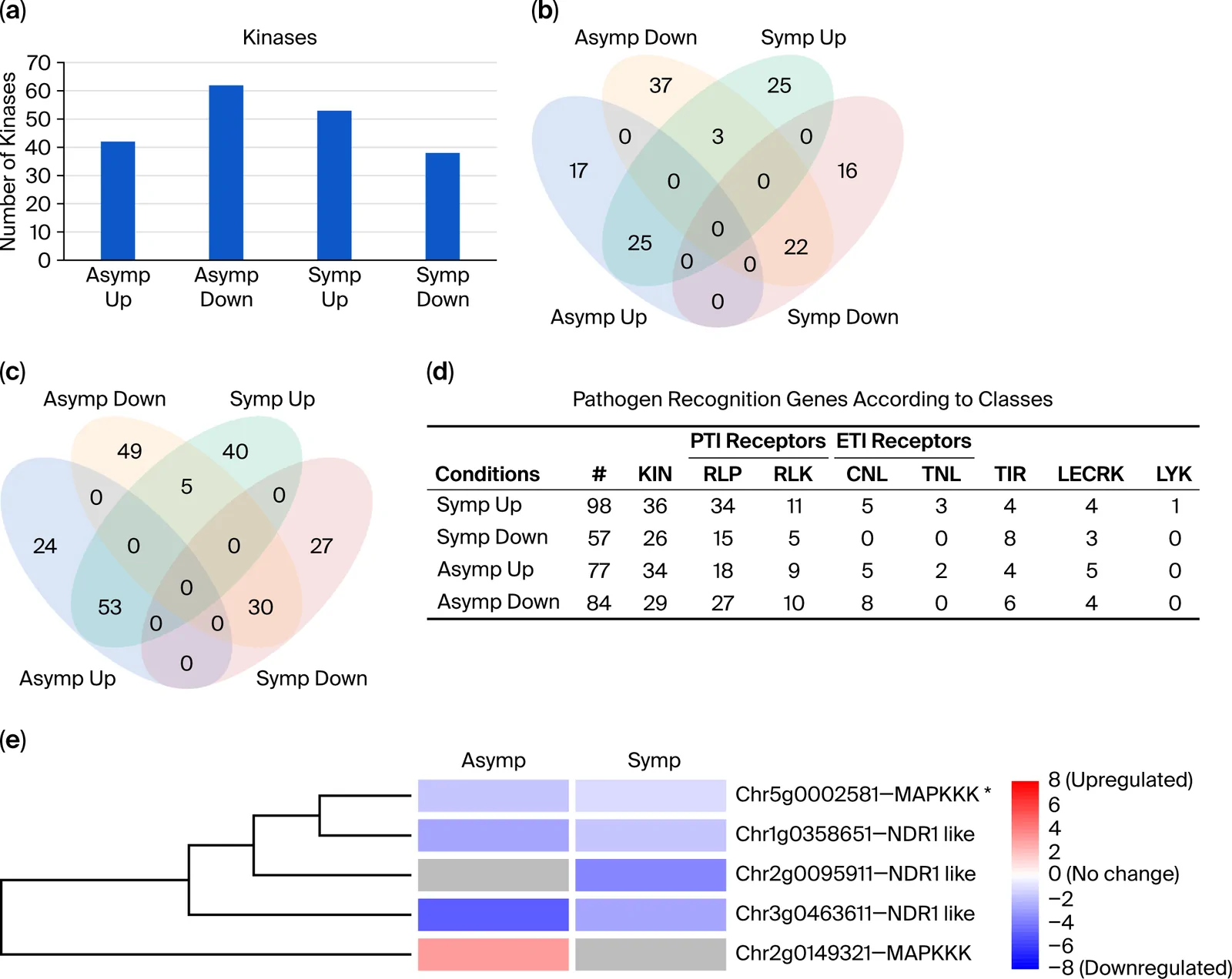
Distribution of kinases and pathogen recognition genes that are elevated or reduced in asymptomatic or symptomatic tissues. (**a**) Bar chart shows the number of kinase genes whose transcripts are upregulated (up) or downregulated (down) in asymptomatic and symptomatic tissues, relative to healthy controls. (**b**) Venn diagram shows differentially expressed kinase genes that are unique to each condition as well as shared between both. (**c**) Venn diagram shows differentially expressed pathogen recognition genes that are unique to each condition as well as shared between both. Colors used in Venn diagrams distinguish categories of genes. (**d**) Classification of pathogen recognition genes according to their involvement in pathogen-triggered immunity (PTI), effector-triggered immunity (ETI), or four additional categories. Abbreviations: Kin, kinase; RLP is receptor like protein; RLK is receptor-like kinase; CNL is coiled–coiled nucleotide binding leucine rich repeat; TNL is Tol–interleukin nucleotide binding leucine rich repeat; TIR is Tol–interleukin receptor; LECRK is lectin receptor like kinase; LYK is lysine-motif receptor kinase. (**e**) Heatmap showing log2FC (*p*adj < 0.05) for four MAPKKK and NDR1-like genes. The color scale (right) indicates the magnitude and direction of log2FC values, where red boxes denote increased transcript levels and blue boxes denote decreased transcript levels compared with healthy tissues The color scale (right) indicates the magnitude and direction of log2FC values, where red boxes denote increased transcript levels and blue boxes denote decreased transcript levels compared with healthy tissues. An asterisk (*) indicates the MAPKKK gene that is also shown in Figure 6b.

Because many plant viruses co-opt host kinases for successful replication and spread, and protein phosphorylation is a central post-translational regulatory mechanism, we systematically examined the expression patterns of kinases in the transcriptome dataset [11,62,63,64]. We also hypothesized that in susceptible hosts, cellular kinases contributing to plant immunity and antiviral defenses may be reduced. Protein kinase genes were aggregated using Gene Descriptions in the GDR database, together with GO terms, InterPro annotations, and enzyme names. Asymptomatic tissues had 42 upregulated kinases, while symptomatic tissues had 53, including 17 unique to asymptomatic, 25 unique to symptomatic, and 25 common to both conditions (Figure 4a,b). Conversely, 62 kinases were lower in asymptomatic and 38 in symptomatic tissues, with 37 unique to asymptomatic, 16 to symptomatic, and 22 common to both (Figure 4a,b). Among these kinases, none were known to directly support replication or cell-to-cell movement of plant-infecting negative-strand RNA viruses. Many encoded protein serine/threonine kinases (STKs), a class that includes known antiviral factors [64]. Notably, three genes (Chr5g0021981 and Chr5g0005131, and Chr2g0120661) were oppositely expressed between conditions, suggesting tissue-specific modulation of defense-related protein kinases (Supplementary Table S6). Together, these patterns indicate that RRV broadly perturbs kinase networks.

To identify kinases associated with canonical resistance pathways and to dissect immune pathway engagement, the bulk transcriptome dataset was analyzed with the DRAGO 3 (Disease Resistance Analysis and Gene Orthology) pipeline from the Plant Resistance Gene database (PRGdb v. 4.0) (Figure 4c,d, and Table S8). A total of 228 factors were identified, representing eight classes of pathogen resistance genes (PRGs) defined by protein domain architecture. Among the upregulated genes, 24 were unique kinases in asymptomatic tissues, 40 were unique to symptomatic tissues, and 53 were common to both conditions. Among the downregulated genes, 49 were unique to asymptomatic, 27 were unique to symptomatic, and 30 were common to both conditions. Only five kinases were oppositely regulated in asymptomatic and symptomatic tissues (Figure 4c). We grouped PRG classes among those associated with effector-triggered immunity (ETI), pattern-triggered immunity (PTI), and other kinases (Figure 4d). Among the upregulated ETI receptor kinases were CNL-type factors identified by NCBI BLAST (https://blast.ncbi.nlm.nih.gov, accessed on 1 July 2026) homology searches as RPM1-like, RPS3-like, RPP13-like, or EXO70G1-like factors (Table S8) which are primarily known for mediating resistance to fungi, oomycetes, or bacterial pathogens and not associated with antiviral resistance. Five TNL factors were upregulated in RRV-infected tissues, and as a class, these can be involved in antiviral resistance in plants. However, homology search results did not reveal any known antiviral TNLs (Figure 4d, and Table S8). Eight CNL-type factors were downregulated, and BLAST homology indicated that Chr5g0004531 is related to the well-known antiviral factor HRT/RCY1, whereas Chr1g0366291 and Chr2g0134391 are respectively linked to the hypersensitive cell death-activating *RGA3* and *RGA4* genes [65,66,67,68,69]. Given that the rose plants used in this study were susceptible, it is possible that these downregulated CNL-type factors fit a model whereby antiviral signaling networks may be lower in mature plants. The majority of differentially expressed PRGs can be classified as PTI receptors rather than ETI receptors in RRV-infected tissues. BLAST homology searches combined with AlphaFold analyses did not identify RLK or RLP factors engaged in ETI/PTI crosstalk [70].

NDR1 is a plasma membrane localized mediator of ETI responses influencing SA accumulation, CNL defense gene induction, and hypersensitive cell death. NDR1 can participate in the activation of MAPK cascades associated with ETI and PTI signaling [71]. We identified three *NDR1-like* genes (Chr1g0358651, Chr2g0095911, and Chr3g0463611) among the downregulated genes in RRV-infected tissues. Two *MAPKKK* genes (Chr5g0002581 and Chr2g0149321) were oppositely regulated (Figure 4e). In total, these patterns indicate that RRV broadly perturbs the fidelity of host kinase networks and associated signal transduction. These data indicate that RRV alters the host immune landscape at the cellular level while also influencing systemic resistance in both asymptomatic and symptomatic tissues, respectively [70].

### 3.5. Cell Wall and Plasma Membrane-Associated Kinases in RRV-Infected Tissues

**Figure 5:**
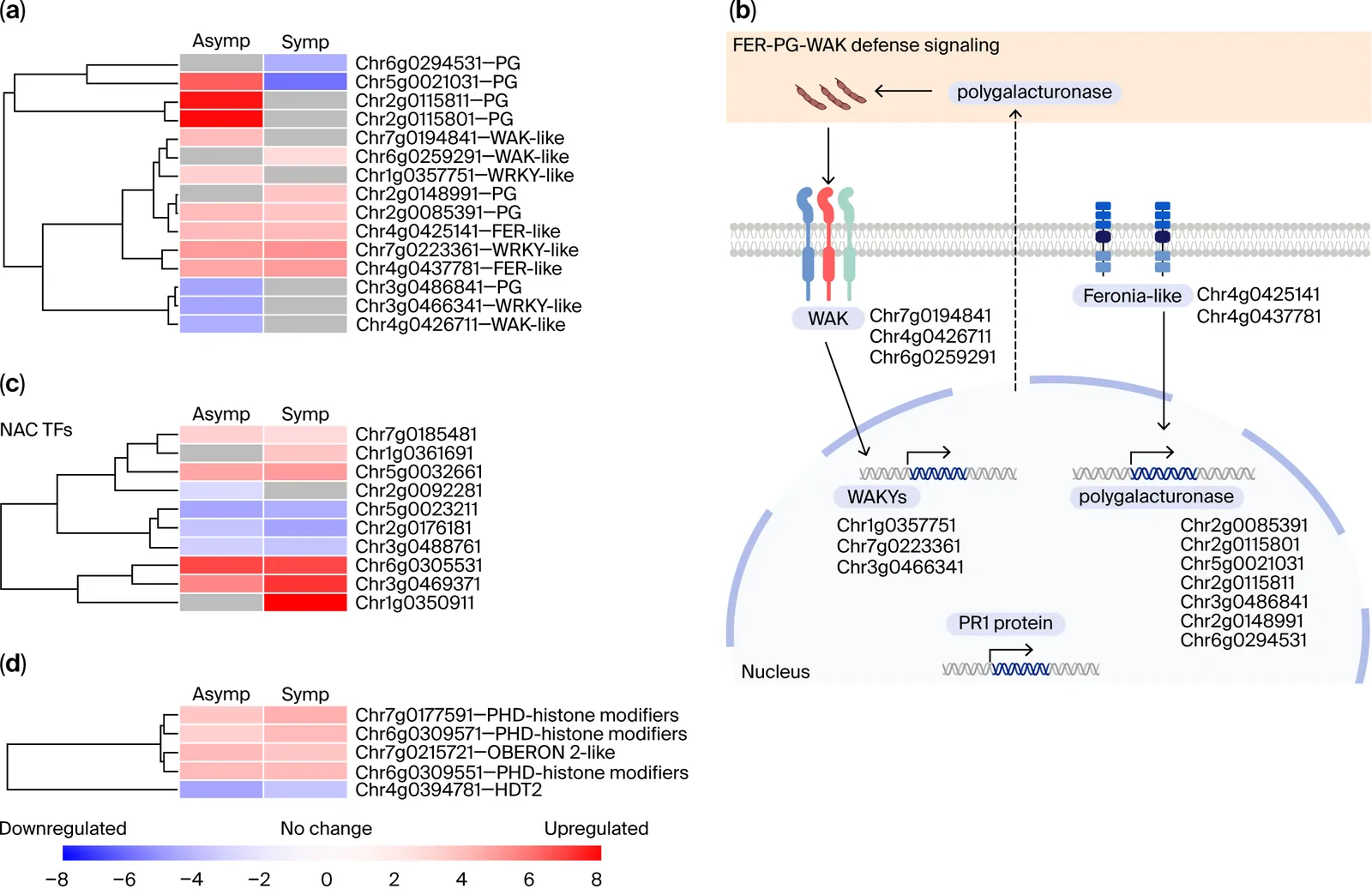
Cell wall- and plasma membrane-associated pathogen recognition factors, chromatic modifying genes, and transcription factors that are differentially expressed in asymptomatic and symptomatic tissues. (**a**,**c**,**d**) Heatmaps representing log2FC (adjusted *p* value, *p*adj < 0.05) relative to healthy tissues, for asymptomatic (Asymp) and symptomatic (Symp) samples. Only genes with statistically significant changes (*p*adj < 0.05) are shown. Each column represents a condition (Asymp or Symp), and each row represents one gene, with gene names listed on the right. The color scale (right) indicates the magnitude and direction of log2FC values, where red boxes denote increased transcript levels and blue boxes denote decreased transcript levels compared with healthy tissues. Three heatmaps combine for: (**a**) wall-associated kinases (WAK-like), polygalacturonases (PGs), two Feronia-like (FER-like), a single WRKY-like transcription factor; (**c**) NAC transcription factors; and (**d**) histone modifiers (PHD–histone modifier, OBERON 2-like, and HDT2). Full gene descriptions and detailed statistics are provided in Tables S2–S6. (**b**) Illustration represents the differentially expressed genes associated with the FER-PG-WAK cell wall defense pathway. Pathogen- or damage-induced PGs generate oligogalacturonides that are sensed by WAKs, which transmit signals to the receptor-like kinase FERONIA (FER) at the plasma membrane. FER-WAK signaling integrates cell wall cues to activate WRKY transcription factors while coordinating PGs and PR1 gene expression to fine-tune cell wall-derived defense signals.

The wall-associated kinases (WAKs), polygalacturonases (PGs), and FERONIA (FER)-type kinases are central components of cell wall integrity and wall-derived immune signaling that can activate Ca^2+^, ROS, and MAPK cascades converging on SA- and JA-related defenses upstream of classic defense regulators, including WRKY and NAC transcription factors. Furthermore, some WRKYs and NACs contribute to cell wall-based defenses, cell wall biosynthesis, and responses to plant virus infection [72,73,74,75,76,77]. Three *WAK*s showed contrasting expression patterns (Figure 5a,b): Chr7g0194841 was upregulated in asymptomatic tissues (log_2_FC = 2.0), Chr6g0259291 was upregulated in symptomatic tissues (log_2_FC = 1.2), whereas Chr4g0426711 was downregulated in asymptomatic tissues (log_2_FC = 2.3). Two *FER-like kinases*, Chr4g0425141 and Chr4g0437781, were induced (log_2_FC ≈ 2.0) in both symptomatic and asymptomatic tissues, suggesting that high and low virus conditions influence FER-mediated signaling and cell wall integrity (Figure 5a,b). Genes encoding PGs also showed contrasting expression patterns: Chr2g0085391 was elevated in both tissues (log_2_FC ≈ 1.6 to 2.0), Chr6g0294531 was markedly lower only in symptomatic tissues (log_2_FC = −2.3), and Chr3g0486841 was markedly lower in asymptomatic tissues (log_2_FC = −2.6). Oppositely regulated PGs included Chr5g0021031, which was substantially elevated in symptomatic tissues (log_2_FC = 4.8) and markedly reduced in asymptomatic tissues (log_2_FC = −4.2). Chr2g0115811 and Chr2g0115801 were uniquely elevated (log_2_FC = 7.1 and 7.7, respectively) (Figure 5a). Together, these coordinated changes in WAK, FER, and PG expression indicated substantial reprogramming of cell wall-associated defense signaling modules during RRV infection, potentially influencing pathogen perception and systemic signaling (Figure 5a,b, and Tables S2–S6).

Three *WRKY* genes were identified among the DEGs (Figure 5a,b): Chr1g0357751 was elevated in asymptomatic tissues (log_2_FC = 1.4), Chr7g0223361 was substantially elevated in both symptomatic and asymptomatic tissues (log_2_FC = 3.4 and 3.1 respectively), and Chr3g0466341 was significantly lower in asymptomatic tissues (log2FC ≈ −2.8) but not obviously changed in symptomatic tissues (Figure 5a,b and Tables S2–S6). NAC factors can act as positive and negative regulators of immunity and sit at the crossroads of SA and JA circuits [74]. Ten *NAC* genes were differentially expressed with six elevated (Chr1g0350911, Chr1g0361691, Chr3g0469371, Chr5g0032661, Chr6g0305531, Chr7g0185481) and four reduced (Chr2g0092281, Chr5g0023211, Chr2g0176181, Chr3g0488761) (Figure 5c).

Epigenetic regulators acting upstream of WRKY and NAC factors include histone and chromatin modifiers (histone acetyltransferases, deacetylases, methyltransferases, and reader proteins with PHD domains) that shape accessibility and expression of antiviral and other pathogen defense genes [78,79,80]. Viruses are known to target the chromatin-transcription factor axis as part of a counter-defense strategy. We identified three *PHD* modifiers (Chr7g0117591, Chr6g0309571, and Chr6g0309551) and an OBERON2 gene (Chr7g0215721) which were substantially elevated in both tissues, whereas the histone deacetylase, *HDT2* (Chr4g0394781) was lower in both tissues (Figure 5d). These patterns suggest that RRV infection triggers selective chromatin remodeling, potentially influencing WRKY- and NAC-mediated transcriptional control of defense genes and epigenetic responses to virus infection.

### 3.6. Gibberellin-Enriched Cis-Regulatory Elements in the Promoters of Key Immune Regulators

The above differential gene expression analysis revealed a subset of established immune signaling hubs (NDR1 and MAPKKKs), master defense transcription factors (WRKYs and NACs), and essential chromatin modifiers (PHD-histone modifiers, OBERON2, and HDT2). To generate hypotheses about potential hormonal and transcriptional control of these 23 genes, we examined predicted *cis*-regulatory elements (CREs) in their promoter regions (Table 1 and Figure S4). This in silico promoter analysis indicated an over-enriched representation of putative gibberellin (GA)-responsive CREs in several key signaling regulators, including two *MAPKKK* genes (Chr5g002581 and Chr2g0149321), *NDR1*, *WRKY*, *NAC*, and chromatin modifier loci (Table 1). Across the 23 upstream sequences analyzed, predicted GA-responsive elements were most frequent, followed by SA-, auxin-, and cytokinin-associated motifs, whereas canonical JA-responsive motifs were not detected. Although these motif patterns are consistent with the metabolite data showing elevated GA levels in asymptomatic tissues reported by Mutethia et al., (2025), they should be interpreted as predictive rather than proven indications of possible hormone regulation [11].

Specifically, Chr5g002581, an ortholog of *MAPKKK18* that has been linked to ABA, stress, growth, and senescence in other systems [81,82,83], contained predicted GA-, auxin-, ABA-, and cytokinin-responsive cis-regulatory elements (CREs) and showed markedly lower transcript abundance in asymptomatic tissues (Figure 4 and Figure S4, Table 1). In contrast, Chr2g0149321, another MAPKKK gene presented in Figure 4, contained predicted GA-, SA-, and auxin-responsive motifs, and exhibited an expression pattern consistent with a potential role in defense (Table 1 and Figure S4). This enrichment of predicted GA-responsive CREs among this conserved subset of candidate host defense genes, together with the scarcity of predicted SA-responsive elements, identifies GA-associated transcriptional regulation as a hypothesis for further investigation. These in silico observations do not demonstrate per se transcription factor binding or functional interactions between GA and classical immune pathways. However, they suggest that NDR1, NAC, WRKY, MAPKKK, PHD-histone modifiers, OBERON2, and HDT2 may be useful candidates for testing whether growth- and defense-associated signaling networks intersect with RRV infection.

### 3.7. Intersecting Differentially Expressed Resistance Gene Candidates with QTL Resistance in Roses

**Figure 6:**
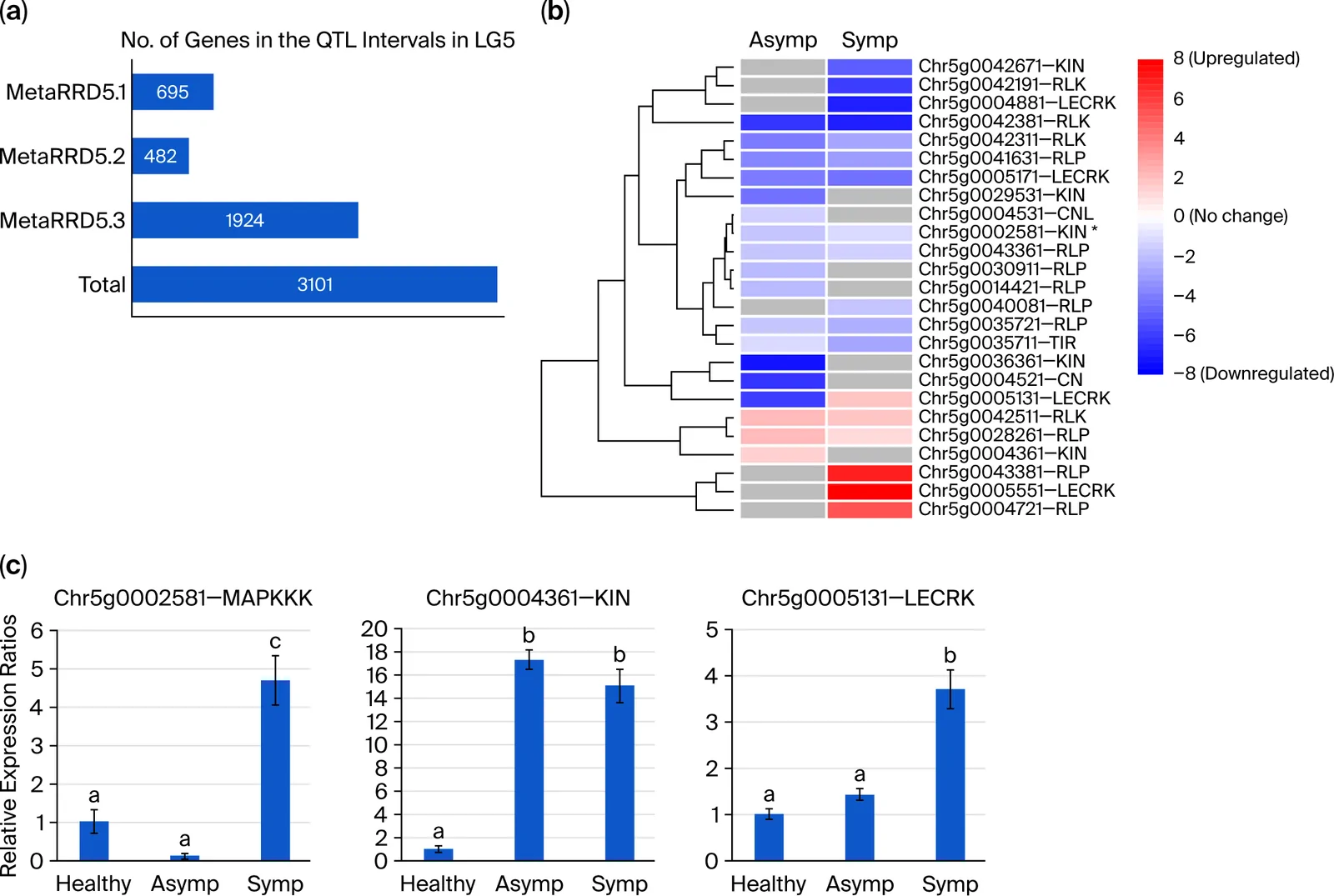
Pathogen recognition genes (PRGs) located in the quantitative trait loci (QTL) in linkage group 5 (LG5). (**a**) The number of genes in three defined Meta-QTLs in LG5 region and the total number of genes represented by these three intervals [16]. (**b**) Expression profiles of differentially expressed PGRs in the Meta-QTLs. Heatmap represents the log_2_FC with adjusted *p* values (*p*adj < 0.05). The color scale (right) indicates the magnitude and direction of log2FC values, where red boxes denote increased transcript levels and blue boxes denote decreased transcript levels compared with healthy tissues. (**c**) RT-qPCR outcomes representing relative changes in gene expression for three genes PRGs using pooled RNA from naturally infected samples. Infection conditions sharing the same lowercase letter indicate no statistically significant difference, whereas differing letters denote significance at *p* < 0.05. An asterisk (*) indicates the Chr5g0002581 gene is also shown in Figure 4e.

To connect transcriptomic responses with genetic loci associated with RRD resistance, we identified differentially expressed genes located within three linkage group 5 (LG5) meta-QTL intervals which we extracted from the Rose Genome Database (RGDR). The three intervals contain a total of 3101 genes: 695 genes in MetaRRD5.1 (1.235–6.005 Mbp), 482 genes in MetaRRD5.2 (9.22–13.05 Mbp), and 1924 genes in MetaRRD5.3 (19.7–40.51 Mbp) (Figure 6a). Across the three LG5 meta-QTL intervals, 200 genes were both QTL-localized and differentially expressed. In asymptomatic tissues, 47 were upregulated and 50 were downregulated; in symptomatic tissues, 57 were upregulated and 46 genes were downregulated (Table 2 and Table S6). These overlapping genes provided biologically informed set of candidate genes for targeted expression analysis in inoculated greenhouse plants.

A primary objective was to determine whether transcript changes identified in field-derived transcriptome data could be measured reproducibly in young, greenhouse-grown rooted cuttings inoculated with an RRV-infectious clone. This required developing an RT-qPCR workflow suitable for low-abundance transcripts and potentially variable RNA quality from rose tissues. Initial assays were performed using cDNA from field-grown plants used for RNA-seq investigations. Initial assays indicated that both target and candidate reference transcripts were present at low abundance. Accordingly, amplification efficiencies were evaluated using a 2-fold dilution which produced closely spaced datapoints within a detectable range of these samples. Assay performance was evaluated with standard curves measuring the slope, R^2^, and efficiency (Table S9). Three primer sets met the criteria for relative quantification and were analyzed using the efficiency corrected Pfaffl method [84,85]. Two moderately induced candidate genes within the LG5 intervals, *Chr5g0005131* (LECRK) and *Chr5g0004361* (KIN), showed 1.4- and 7.7-fold higher expression in RRV-infected tissues than in healthy controls (Figure 6c). *Chr5g0002581* (MAPKKK) was lower in asymptomatic tissue, consistent with the transcriptome dataset, but was unexpectedly induced approximately 3-fold in symptomatic tissue relative to healthy controls. These results support the feasibility of measuring selected LG5 candidate genes in greenhouse material while also suggesting that transcript responses may depend on disease stage or tissue symptom status.

We next evaluated the same candidate genes in RRV-infectious clone-inoculated young plants (var. Double Knockout) maintained in the greenhouse. RNA was collected from plants at 3, 6, and 9 weeks post inoculation. Although each candidate gene showed induction relative to the healthy controls, transcript accumulation varied substantially among inoculated plants across time points (Figure S4). This variability likely reflects biological heterogeneity in the infection process and substantial plant-to-plant variation.

To refine resistance candidates within the QTL region that might be linked to RRV infection, we integrated QTL localization, DRAGO-based resistance gene prediction, and RNA-seq data, yielding 25 candidate genes (Figure 6b; Table S8). Considering only the asymptomatic tissues with low virus titer, only three were obviously upregulated (log_2_FC ≈ 2.0): Chr5g0042511 (RLK), Chr5g0028261 (RLP), and Chr5g0004361 (KIN), whereas the remaining candidate genes were moderately or substantially lower (log_2_FC ≈ −2.0 to −6.0). In symptomatic tissues with high virus levels, six genes exhibited much higher levels of expression (log_2_FC ≈ 2.0 to 8.0): Chr5g0005131 (LECRK), Chr5g0042511 (RLK), Chr5g0028261 (RLP), Chr5g0043381 (RLP), Chr5g0005551 (LECRK), and Chr5g0004721 (RLP), while the others were substantially downregulated (log2FC range from −2.0 to −8.0) (Figure 6b). Notably, Chr5g002581, previously highlighted as a MAPKKK in Figure 4, is also located within the LG5 meta-QTL interval, making it a particularly compelling candidate defense regulator within QTL5.

## 4. Discussion

Integrated metabolite and defense gene analysis revealed that rosetted and asymptomatic tissues collected from the same naturally RRV-infected rose plants were associated with distinct SA, JA, and oxylipin profiles. This tissue-specific pattern is consistent with the uneven distribution of virus accumulation [11] and likely vector feeding expected within large, structurally complex woody shrubs. Rosetted tissues with higher virus titers and asymptomatic tissues with lower virus titers reflect locally distinct infection environments, whereas responses shared across tissues may reflect systemic signaling within the plant. Importantly, the observed metabolite and transcript patterns do not establish whether these defense-associated states control virus accumulation or symptom development. Instead, they identify candidate signaling networks that may contribute to tissue-specific defense responses during natural RRV infection and provide priorities for targeted functional validation.

Because these plants were naturally infected by an insect-transmitted virus, we anticipated that combined vector/virus pressures would perturb the normally antagonistic SA/JA balance described in susceptible hosts [86]. Compared to the symptomatic tissues, the asymptomatic tissues had higher bioactive JA conjugates and AzA, although marginally higher than in healthy tissues. Asymptomatic tissues also had lower SA levels, lower expression of SABP2 and NPR1, and robust expression of PR1. This combined metabolite and transcript pattern indicates a non-uniform defense-associated state in which higher PR1 expression and some JA- and AzA-associated responses may occur alongside lower SA abundance and lower expression of key SA-signaling components. This interpretation is consistent with reports of context-dependent antagonism and occasional synergy between SA and JA signaling reported in other systems [86]; but we note that functional experiments will be required to test whether these patterns directly contribute to symptom outcomes.

Differentially expressed genes were enriched in stress-responsive GO categories, including genes associated with SA, JA, and oxylipin biosynthesis and signaling, as well as cell wall and plasma membrane-related defense processes. The lower expression of *SABP2*, *NPR1* and several *PR* genes, together with increased PR1 expression, is compatible with attenuated SA-associated signal transduction rather than uniform activation of a canonical SA-defense program. This pattern is also consistent with reports that JA and oxylipin signaling can interact antagonistically or synergistically with SA pathways in a context-dependent manner. Figure 1 showed altered levels of 12-OPDA, which is both a precursor to JA and a bioactive defense molecule that operates autonomously to intersect with systemic defense priming [43,87,88]. Its lower abundance in asymptomatic tissues does not establish virus-mediated suppression of jasmonate-related defenses [89].

We initially hypothesized that key intermediates and transcriptional regulators in our dataset contain JA-responsive cis elements that recruit trans-repressors, thereby antagonizing SA activation even as PR1 remains elevated [53,90]. To our surprise, we did not detect canonical JA-responsive cis elements in the promoters of key regulatory genes, but instead found an abundance of GA-, followed by SA- and ABA-responsive motifs. These in silico suggest alternative candidate models in which GA-, SA-, and ABA-associated signals may be linked to defense gene expression patterns. Because transcription factor binding site presence alone does not demonstrate functional regulation, these results should be viewed as hypotheses about potential regulatory nodes rather than evidence of direct hormonal control. Future work will need to test whether GA signaling components (e.g., DELLA proteins) interact with SA pathways to modulate systemic resistance and whether genes in MetaRRD5.3 contribute to defense-associated expression, virus accumulation, or symptom development. Functional assays of promoter activity and controlled hormone or inhibitor experiments will be essential to evaluate these hypotheses.

SAR relies on SA accumulation, MeSA transport, and its hydrolysis by SABP2 to regenerate defense-active SA in distal tissues. In our asymptomatic tissues, we observed low SA and low SABP2 expression together with higher AzA, which is a mobile chemical signal that moves systemically to contribute to systemic resistance against biotrophs [87,90,91,92]. The combination of elevated AzA, lower virus loads, and higher PR1 expression is consistent with a defense-primed state predisposed to stronger SA-responsive gene expression upon appropriate triggers [93,94]. Notably, other research has shown that exogenously applied SA and caffeic acid (CFA) derivatives can stimulate SAR and exhibit antiviral activities [40]. In Mutethia et al. (2025) [11], we reported higher endogenous CFA levels and biosynthetic genes in symptomatic than asymptomatic tissues, while CFA levels in asymptomatic tissues were lower than in healthy tissues. Because CFA is an intermediate in the phenylpropanoid pathway that also produces SA and can influence flux toward SA and SA signaling, these patterns may help explain differences in virus accumulation between tissue types. However, we emphasize that these associations do not demonstrate that CFA and AzA directly restrict virus movement; targeted experiments will be required to establish causation.

Three meta-QTLs for Rose Rosette Disease (RRD) resistance on linkage group 5 (LG5), MetaRRD5.1 (1.235–6.005 Mbp), MetaRRD5.2 (9.22–13.05 Mbp), and MetaRRD5.3 (19.7–40.51 Mbp) provide genomic anchors for interpreting transcriptional changes [16]. MetaRRD5.1 and MetaRRD5.2 each contain 29 differentially expressed genes with distinct up/down patterns across asymptomatic and symptomatic tissues, while MetaRRD5.3 contains 142 differentially expressed genes, representing a broader regulatory landscape, possibly encompassing defense and signaling hubs [16]. Beyond LG5, we observed an overrepresentation of downregulated genes on chromosomes 2 and 5, aligning with prior susceptibility/resistance QTLs mapped to these chromosomes, and compatible with broad repression of loci that may contribute to antiviral barriers [13,15,16,95]. Nevertheless, these interferences are correlative and must be interpreted cautiously given differences among cultivars and the uncertainty in precise mapping QTL intervals and causal genes.

## 5. Conclusions

In summary, the observed changes in phytohormone and oxylipin signaling in naturally infected roses resemble a mixture of adaptive immune strategies and viral interference. Asymptomatic tissues display a pattern consistent with a defense-primed state marked by elevated AzA and PR1 expression alongside lower SA biosynthesis and suppression of key signaling components such as SABP2 and NPR1. This pattern points toward partially activated SAR-like pathways influenced by AzA and 12-OPDA signals rather than bulk SA accumulation. Promoter motif analysis indicates GA-SA crosstalk might influence systemic resistance to virus infection. Symptomatic tissues, which carry greater virus loads, are associated with stronger CFA and phenylpropanoid, altered oxylipin profiles, and changes in defense-related GO categories, suggesting that multiple hormone pathways and gene networks may be required under high virus pressure. Within this correlative framework, LG5 meta-QTL regions, particularly MetaRRD5.3, emerge as candidate regulatory hubs integrating hormone signaling and defense gene expression.

Together, these insights advance new hypotheses of how virus and vector pressures rewire hormone and gene expression networks and provide a foundation for breeding strategies and chemical interventions to enhance systemic resistance. Future work should focus on dissecting GA–SA interactions, validating motif functionality, and testing the roles of specific oxylipins in antiviral defense and symptom modulation.

## Figures and Tables

**Table 1:** Numbers of CREs associated with phytohormone regulation in defense-related genes.

Locus	Gene Name	GA	SA	Auxin	ABA	Cytokinin	JA
Chr2g0095911	NDR1 like1	17	6	3	2	1	0
Chr3g0469371	NAC TF	16	3	2	2	1	0
Chr1g0361691	NAC TF	16	5	3	1	0	0
Chr5g0032661	NAC TF	12	1	2	2	0	0
Chr3g0463611	NDR1-like 10	12	3	2	1	0	0
Chr3g0466341	WRKY49-like	11	2	1	0	0	0
Chr6g0309571	PHD–histone modifier	11	4	2	0	1	0
Chr6g0305531	NAC TF	10	3	3	0	0	0
Chr2g0176181	NAC TF	10	4	2	2	0	0
Chr2g0149321	MAPKKK-like	10	5	5	0	1	0
Chr7g0215721	OBERON 2	9	3	4	0	1	0
Chr6g0309551	PHD–histone modifier	9	3	6	1	0	0
Chr5g0002581	MAPKKK-like18	8	1	3	3	2	0
Chr5g0023211	NAC TF	8	1	4	0	1	0
Chr7g0223361	WRKY9	8	4	3	1	0	0
Chr7g0177591	PHD–histone modifier	8	5	4	2	0	0
Chr1g0358651	NDR1 like 2	7	2	7	0	0	0
Chr3g0488761	NAC TF	7	3	2	4	1	0
Chr7g0185481	NAC TF	7	3	3	3	0	0
Chr1g0350911	NAC TF	7	6	4	3	1	0
Chr2g0092281	NAC TF	6	2	2	2	5	0
Chr4g0394781	HDT2	6	2	2	0	1	0
Chr1g0357751	WRKY24-like	2	1	2	4	1	0

**Table 2:** Number of differentially expressed genes in linkage group 5 (LG5).

QTLs on LG5	Total No. Genes in Interval	Up in Asymptomatic	Down in Asymptomatic	Up in Symptomatic	Down in Symptomatic
MetaRRD5.1	695	4	9	10	6
MetaRRD5.2	482	7	9	10	3
MetaRRD5.3	1924	35	32	37	37
Total	3101	46	50	57	47

The meta-QTLs in the linkage group 5 (LG5) were reported in Hochhaus et al. (2023) [16].

## Data Availability

The datasets generated during this study are available at the NCBI SRA (PRJNA1285908). All other data is available on request.

## Supplementary Materials

The following supporting information can be downloaded at https://www.mdpi.com/article/10.3390/v18080906/s1, Figure S1. Diagrammatic representation of workflows for (A) metabolite analysis and (B) bioinformatic studies of RNA-seq datasets; Figure S2. Heatmap visualization of systemic hormone-related metabolic shifts; Figure S3. Bar plot showing Gene Ontology (GO) enrichment terms by level 3, of differentially expressed genes (DEGs) related to plant defense and stress responses under asymptomatic and symptomatic tissue (Tables S2 and S6); Figure S4. Depiction of hormone responsive promoter elements in the upstream 1000 bp for 23 genes; Figure S5. RT-qPCR analysis of RRV-infectious clone-inoculated rose plants; Table S1: List of metaboltes and phytohormone and their full name; Tables S2: Rosa chinensis genes that are upregulated in RRV infected, asymptomatic leaves relative to healthy leaves; Tables S3: Rosa chinensis genes that are downregulated in RRV infected, asymptomatic leaves relative to healthy leaves; Tables S4: Rosa chinensis genes that are upregulated in RRV infected, symptomatic leaves relative to healthy leaves; Tables S5: Rosa chinensis genes that are downregulated in RRV infected, symptomatic leaves relative to healthy leaves; Tables S6: Rosa chinensis genes that are in the upregulated and downregulated datasets in Tables S2 through S5; Table S7: Rosa chinensis genes classified as R genes according to DRAGO and indicators of whether they occur in linkage group 5 (LG5); Table S8: Differentially expressed genes in the QTLs of linkage lroup 5 (LG5); Table S9: Primers used for RT-PCR and RT-qPCR.
