# Supplementary material for: Distinct Activation of Defense-Related Gene Networks in Rosetted Versus Symptomless Shoots in Rose Rosette Virus-Infected Roses
Source: Viruses. 2026 Aug 18;18(8):906. doi: 10.3390/v18080906 (PMC13517598; doi:10.3390/v18080906)
Supplement: Supplementary file 1 [file viruses-18-00906-s001.zip › Supplementary Figures.pdf]

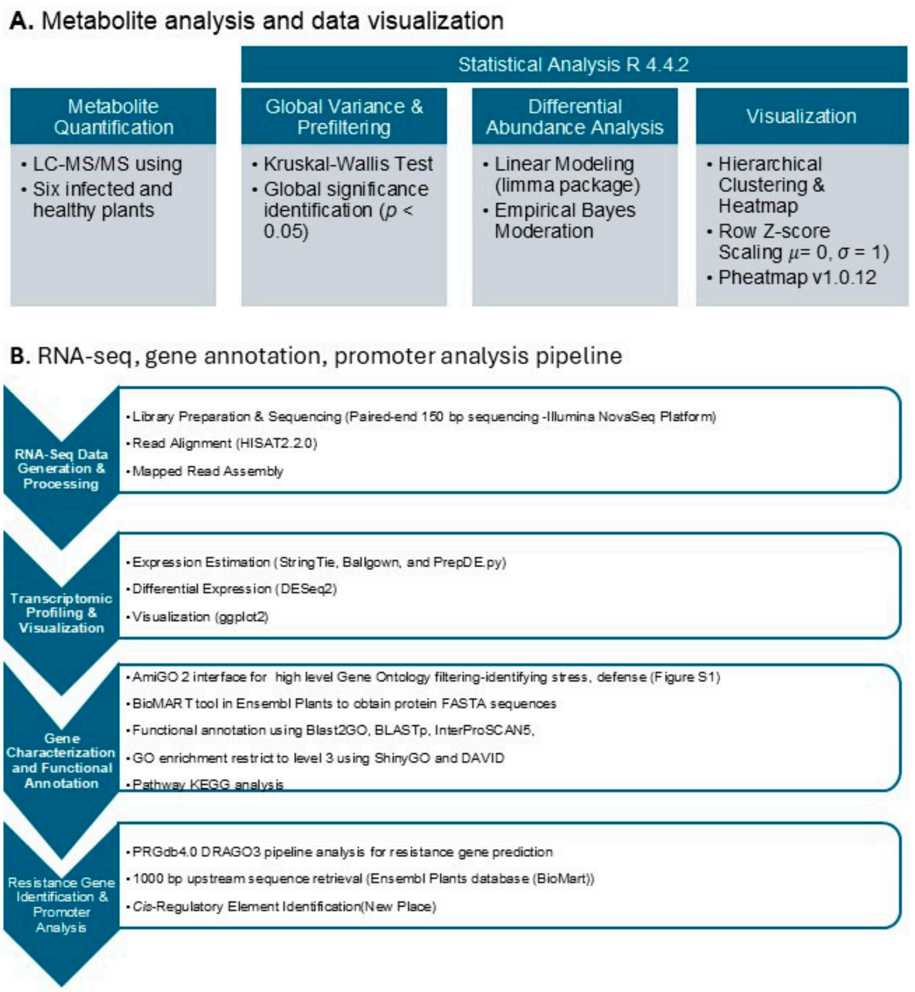

Figure S1. Diagrammatic representation of workflows for (A) metabolite analysis and (B) bioinformatic studies of RNA-seq datasets.

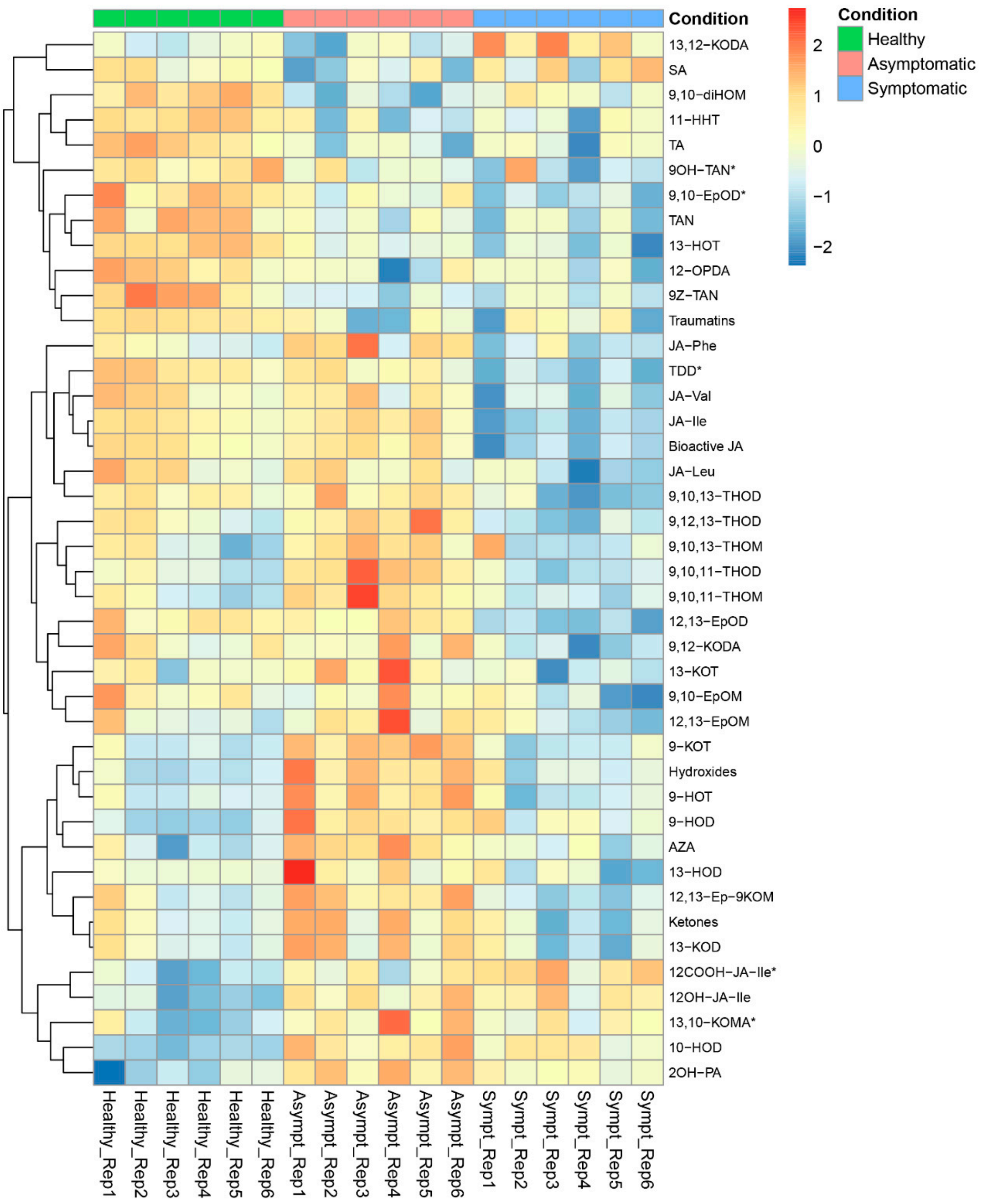

Figure S2. Heatmap visualization of systemic hormone-related metabolic shifts. The heatmap displays the relative abundance of significant metabolites across healthy, asymptomatic , and symptomatic tissue replicates. The log2-transformed expression values were standardized using row-wise Z-scores to allow side-by-side visual comparison of relative changes across metabolites. The blue-to-red color scale indicates lower-to-higher than average relative concentrations. Hierarchical clustering dendrograms on the left highlight identified relationships and similar accumulation patterns between compounds.

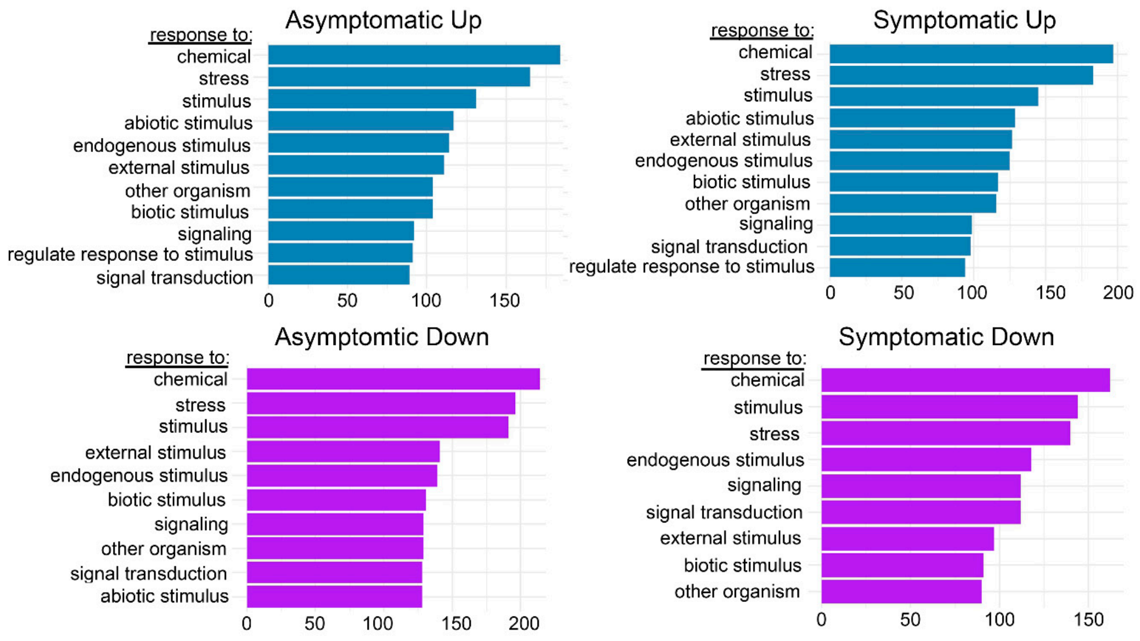

Figure S3. Bar plot showing Gene Ontology (GO) enrichment terms by level 3, of differentially expressed genes (DEGs) related to plant defense and stress responses under asymptomatic and symptomatic tissue (**Tables S2 and S6**). The full GO terms are response to chemical, stress, stimulus, etc are shorted on the Y-axis. The X-axis is the count of the numbers of genes. The blue bars represent the number upregulated genes and the magenta bars represent the number of downregulated genes for each term, when comparing each condition (asymptomatic or symptomatic) to healthy controls.

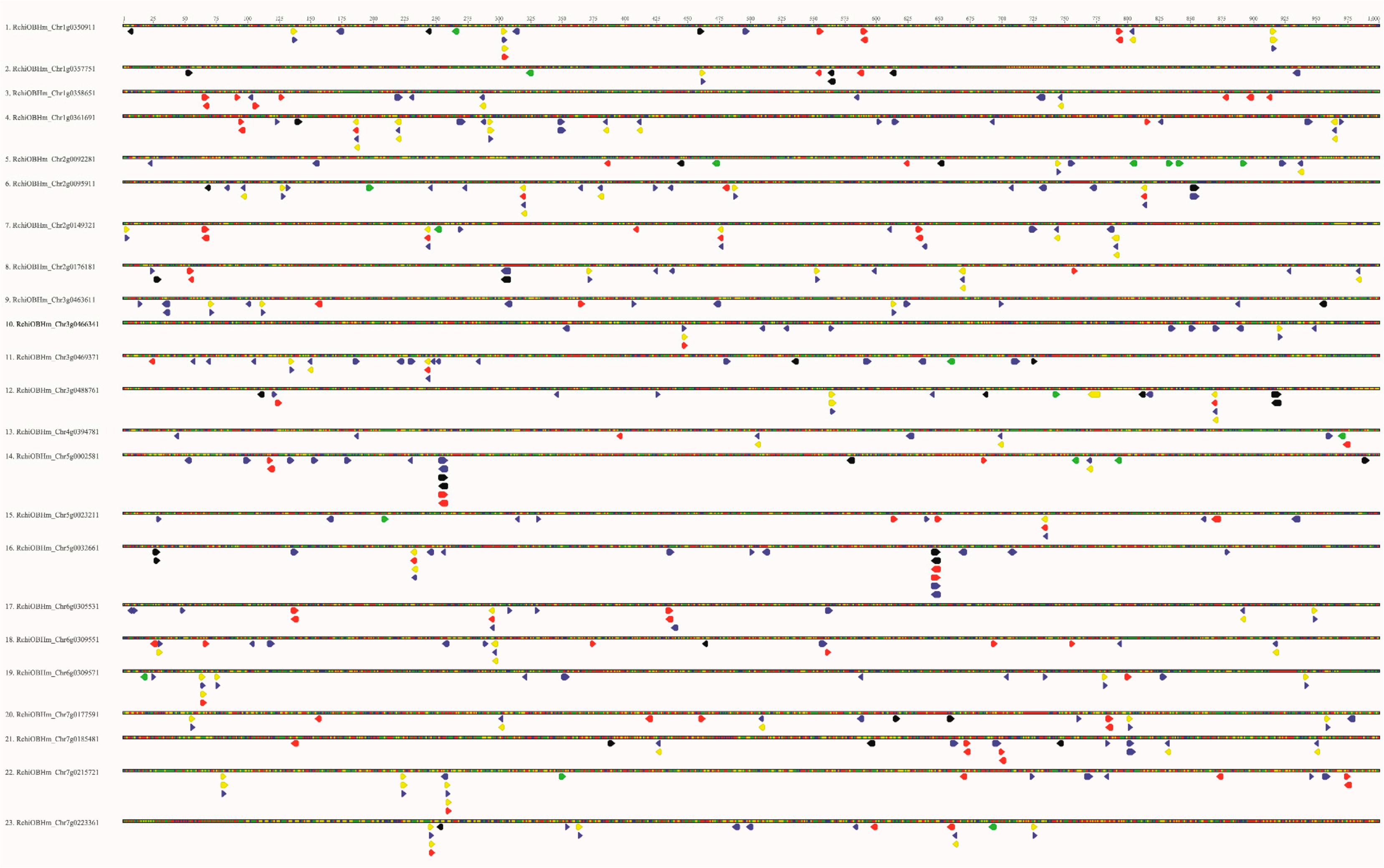

Figure S4. Depiction of hormone responsive promoter elements in the upstream 1000 bp for 23 genes. Genes are listed in the left. Colored arrowheads identify elements: black = ABA, green = cytokinin, blue = GA, yellow = SA, and red = auxin.

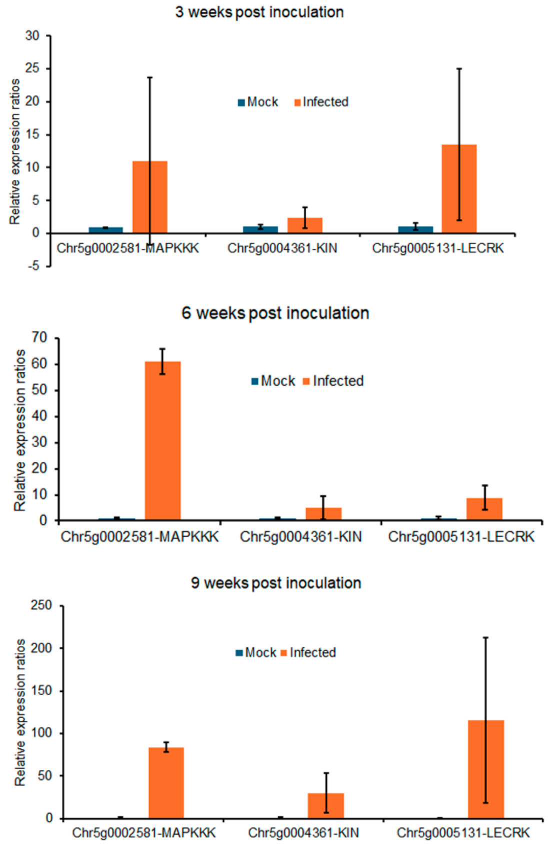

Figure S5. RT-qPCR analysis of RRV-infectious clone inoculated rose plants. The relative changes in gene expression for three genes PRGs was obtained from individual plants showed wide ranging results, although a general trend that suggests virus-induced gene expression. Statistical analysis failed to identify differences between healthy and infected individuals due to the wide-ranging data.
